# Underestimated prevalence of heart failure in hospital inpatients: a comparison of ICD codes and discharge letter information

**DOI:** 10.1007/s00392-018-1245-z

**Published:** 2018-04-17

**Authors:** Mathias Kaspar, Georg Fette, Gülmisal Güder, Lea Seidlmayer, Maximilian Ertl, Georg Dietrich, Helmut Greger, Frank Puppe, Stefan Störk

**Affiliations:** 1 Comprehensive Heart Failure Center (CHFC), Department of Internal Medicine I, Würzburg University Hospital, Am Schwarzenberg 15, 97078 Würzburg, Germany; 2 Chair of Computer Science VI, University of Würzburg, Würzburg, Germany; 3 Service Center Medical Informatics, Würzburg University Hospital, Würzburg, Germany

**Keywords:** Data warehouse, Information extraction, ICD coding, Heart failure, Electronic health records

## Abstract

**Background:**

Heart failure is the predominant cause of hospitalization and amongst the leading causes of death in Germany. However, accurate estimates of prevalence and incidence are lacking. Reported figures originating from different information sources are compromised by factors like economic reasons or documentation quality.

**Methods:**

We implemented a clinical data warehouse that integrates various information sources (structured parameters, plain text, data extracted by natural language processing) and enables reliable approximations to the real number of heart failure patients. Performance of ICD-based diagnosis in detecting heart failure was compared across the years 2000–2015 with (a) advanced definitions based on algorithms that integrate various sources of the hospital information system, and (b) a physician-based reference standard.

**Results:**

Applying these methods for detecting heart failure in inpatients revealed that relying on ICD codes resulted in a marked underestimation of the true prevalence of heart failure, ranging from 44% in the validation dataset to 55% (single year) and 31% (all years) in the overall analysis. Percentages changed over the years, indicating secular changes in coding practice and efficiency. Performance was markedly improved using search and permutation algorithms from the initial expert-specified query (F1 score of 81%) to the computer-optimized query (F1 score of 86%) or, alternatively, optimizing precision or sensitivity depending on the search objective.

**Conclusions:**

Estimating prevalence of heart failure using ICD codes as the sole data source yielded unreliable results. Diagnostic accuracy was markedly improved using dedicated search algorithms. Our approach may be transferred to other hospital information systems.

## Background

Heart failure has become the leading diagnosis at hospital discharge and the most important driver of in-hospital mortality in Germany [1]. These estimates are based on counts provided by hospitals utilizing the German modification of the International Classification of Diseases (ICD)-10. Respective ICD-10 codes identifying heart failure within the German ICD-10 catalogue are I11.*, I13.0, I13.2, and I50.*. However, estimating the true number of patients suffering from heart failure based on this catalogue is unreliable for several reasons. The heart failure syndrome, especially in its early stages, may go unrecognized or may not be encoded as an explicit diagnosis; further, various financial incentives provided by the German health care system drive the likelihood of a specific ICD code entering a patient’s list of discharge diagnoses. These incentives favor the encoding of diagnoses associated with the most favorable reimbursement profile and may therefore considerably affect the “true number” of patients burdened by heart failure. After hospital discharge, however, only the most important diagnoses (e.g., the top three) are reported to and collected by higher level organizations, e.g., health insurances or statutory registries. Thus, the “prevalence” of a certain condition may be augmented or suppressed depending on its re-imbursement profile and subsequent quality of coding. Furthermore, the quality of documentation itself, e.g., staff training [2] and the marked changes imposed on respective workflows (e.g., change from paper-based records to electronic record systems [3]), have a major influence on disease statistics. Despite these shortcomings, the above-mentioned approach of collecting ICD diagnoses remains the prime source for public health decisions [4, 5].

Beside the statutory census of disease statistics, attempts have been made towards a more reliable and comprehensive identification of diagnoses from clinical routine data. Most of them, however, based their algorithm on coded diagnoses [6]. Because of these reasons, a better and earlier recognition of heart failure patients is of utmost importance [7]. Since modern hospitals can provide a wealth of electronic patient-based information, this data may be used to improve or corroborate diagnostic certainty and comprehensiveness.

The objective of the current study was to approximate the “true number” of patients suffering from heart failure at a tertiary care center. Against a physician-based reference standard, we compared the performance of ICD-based diagnosis versus advanced definitions based on algorithms that integrate various sources of the hospital information system. We hypothesized that (a) ICD-based diagnosis may underestimate the true prevalence of heart failure and (b) a catalogue of criteria defining heart failure utilizing various sources of the hospital information system will advance diagnostic accuracy.

## Methods

### The Würzburg data warehouse

The clinical data warehouse (DWH) implemented at the Würzburg University Hospital provides a homogeneous and structured access to pseudonymized data of 100% of the hospital’s patient cases originally stored within various separate information systems (e.g., the central administrative system, electronic patient chart used on wards, and systems for laboratory values and discharge letters). The only current exceptions are data from psychiatric and child care facilities for data protection reasons. The technical set-up is based on open-source systems and has been described elsewhere [8–10]. Data of the DWH can be queried in (a) structured form (e.g., patient demographics, diagnoses as ICD codes, procedures as codes of the German procedure classification “Operationen- und Prozedurenschlüssel” (OPS), and laboratory values); (b) semi-structured form (e.g., echocardiography, cardiac catheterization), and (c) unstructured form (e.g., discharge letters). The most innovative add-on to the DWH is the unique information extraction and ad hoc text search functionality, which allows to create parametrized information from semi- and unstructured reports and to search for any textual item (e.g., search within discharge letters for text combinations including variants and negations or extract numeric parameters from echocardiographic reports) [11].

### Patient selection

The Medical Department I of the Würzburg University Hospital specializes in, but is not limited to, emergency medicine, intensive care, cardiology, pulmonology, nephrology, and endocrinology. For the current analysis, we used all cases of patients treated at the Medical Department I between the years 2000 and 2015 for whom a discharge letter was available.

### Reference standard for the definition of heart failure

A sample of consecutive patients treated at the Medical Department I was drawn from the DWH within a randomly selected period (January 1 to January 31, 2009), yielding 1042 cases. These patients were manually checked by a cardiologist with long-standing experience in the care of heart failure patients (GG). Information used by the physician included ICD codes, the discharge letter, and the echocardiographic report (if available). The physician assigned a label (“heart failure: yes/no”) to each case, which was then used as reference standard for subsequent analyses.

### Algorithms for automated detection of heart failure

In order to investigate heart failure detection algorithms, 18 subqueries of relevant heart failure-related concepts were defined within the user interface of our DWH and presented in the rows of Table 1. Each subquery considers a specific fact and either is a restriction on a numeric DWH parameter (e.g., subquery Echo-EF ≤ 45 represents a left ventricular ejection fraction (LVEF) ≤ 45% captured from echocardiographic reports after information extraction), the existence of an ICD diagnoses (e.g., subquery ICD-Any-HF represents the existence of any heart failure related ICD) or text searches within the discharge letter suggesting presence of heart failure (e.g., subquery Text-Left-HF represents the occurrence of a textual synonym for “left ventricular heart failure”). Text searches were specified to account for typing errors, synonyms and negations [12].

**Table 1:** Automated advanced data warehouse interrogation to detect heart failure

Subquery name^a^	Search terms (including synonyms and word parts in German language)	HF detection algorithms				
		*M*_ICD_	*M*_Expert_	*A*_Precision_	*A*_Sensitivity_	*A*_F1_
Echo-EF ≤ 45	lvef ≤ 45		×	×		×
Echo-EF < 50	lvef < 50					
ICD-Any-HF	I13.2, I13.0, I11, I50 (including more specific diagnosis)	×	×		×	×
Text-Heart-Failure	herzschw* OR herzinsuff*		×	×	×	×
Text-Cardiac-Decompensation	(card| kard| kardiopulmo| cardiopulmo| hydrop| herz| link)* dek*		×		×	×
Text-Systolic-Failure	(card| kard| cardiopulmo| kardiopulmo| hydrop| herz)* pumpvers* OR vorwärtsversag* OR (kard| card)* schock*		×	×	×	×
Text-Dilated-Cardiomyopathy	dilat* (kardiomy| cardiomy)* OR dcm				×	
Text-NYHA	nyha		×		×	
Text-Left-HF	(kard|linksherz)* insuff*		×		×	
Text-Right-HF	(rechtsherz| diast)* insuff*		×			
Text-Reduced-LV-Function	(komp| reduzierte| eingeschränkte| verminderte)* (link| sys)* ventr* funkti* OR (komp| reduzierte| eingeschränkte| verminderte)* lv funkti* OR ventrik* (komp| reduzierte| eingeschränkte |verminderte)* funkti* OR sys* dysfunkti*		×		×	
Text-Reduced-RV-Function	(komp| reduzierte| eingeschränkte| verminderte)* (rechts| dias)* ventr* funkti* OR (komp| reduzierte| eingeschränkte| verminderte)* rv funkti* OR dias* dysfunkti*		×			
Text-Pulmonary-Edema	lung*ödem* OR lung*stau* OR stau*lung*		×			
Text-Left-Ventricular-Hypertrophy	lv hypertr* OR link*ventr* hypertr*				×	
Text-Left-Atrial-Enlargement	(link*vorho*| link*atri*| la) (verg| dilat| hypertr)*				×	
Text-Diastolic-Dysfunction	(komp| eingeschr| vermind)* (ventr| dias)* funkti* OR dias* (dysfunkti*| relax*stör*)				×	
Lab-NT-proBNP ≥ 1000	nt-probnp (pg/mL) ≥ 1000					
Lab-NT-proBNP ≥ 3000	nt-probnp (pg/mL) ≥ 3000					

M_Expert_ indicates the initially defined query by the clinical expert and a computer scientist. *M*_ICD_ indicates the algorithm using sole ICD codes for comparison. *A*_F1_, *A*_Precision_, and *A*_Sensitivity_ indicate search algorithms optimized using permutation testing. Queries use Boolean “OR” operators, which means that each single hit justifies presence of heart failure

*LV* left ventricular, *RV* right ventricular, *EF* ejection fraction, *HF* heart failure, *NYHA* New York Heart Association, *DCM* dilated cardiomyopathy, *NT-proBNP* N-terminal prohormone of brain natriuretic peptide

^a^Sources: *Echo* echocardiography report, *ICD* ICD diagnosis, *Text* unstructured text from discharge letter, *Lab* laboratory value from routine laboratory testing

The algorithms used to detect patients with heart failure (i.e., *M*_ICD_, *M*_Expert_, *A*_Precision_, *A*_Sensitivity_, *A*_F1_) are presented in the right-hand columns of Table 1, each by a selection of subqueries that needed to be combined for the full algorithm. Each hit of any of an algorithm’s subqueries stands for the presence of heart failure. Two of the algorithms were manually specified: *M*_ICD_ indicates an algorithm that solely utilizes ICD codes and *M*_Expert_ indicates an algorithm (i.e., subqueries used for this DWH interrogation) pre-specified by cardiologists based on clinical experience. The other three algorithms (*A*_Precision_, *A*_Sensitivity_, and *A*_F1_) originated from iterative permutation testing utilizing all defined subqueries. They were optimized to yield the most favorable results regarding the chosen measures (i.e., precision, sensitivity, and F1 score; for definitions see “Data analysis”) with regard to the reference standard definition of heart failure described in the previous section. The algorithms were computed utilizing exactly the same data that the physician used to evaluate the reference standard: the discharge letter, the ICD codes, and the echocardiographic report (if available).

### Data analysis

The data for the current analyses were exclusively taken from the DWH via its graphical user interface and the subqueries defined in Table 1. Analysis was done using the software package R [13]. Presence of heart failure was captured based on data of individual hospital visits of individual patients (i.e., one case); each patient was counted once per year. The proportions of true positive, false positive, true negative, and false negative matches were calculated. Further, precision, sensitivity, and the F1 score were computed to provide integrated measures of the accuracy of the match between automated heart failure detection and the reference standard. Precision (also called positive predictive value) describes the share of algorithmically labeled heart failure patients who indeed have heart failure, out of all algorithmically labeled heart failure patients; e.g., a precision of 100% means that all selected patients truly have heart failure. Sensitivity (also called true positive rate or recall) is the share of algorithmically labeled heart failure patients who indeed have heart failure, out of all patients with heart failure; e.g., a sensitivity of 100% means that all patients with heart failure are selected. The F1 score is the harmonic mean of precision and sensitivity and is used as the overall accuracy measure in this analysis; e.g., an F1 score of 100% means that exactly the patients who truly have heart failure are selected and an F1 score of 85% would describe the prevalence of heart failure with an estimated error of 15%. Measures of any permutation of the subqueries were computed in R, utilizing single DWH exports of each subquery, to maximize the F1 score and aiming to yield a precision and sensitivity of at least > 90% but still have a corresponding sensitivity and precision > 60%, respectively. Frequencies and percentages were used to present aggregated data across periods under study.

## Results

From 2000 to 2015, 110,742 individual patients were treated at and received a discharge letter from the Department of Medicine I of the Würzburg University Hospital. Of these patients, 71,625 had at least one inpatient visit. After splitting the 16-year period into four 4-year periods (i.e., 2000–2003, 2004–2007, 2008–2011, and 2012–2015), respective counts for all patients (inpatients) were 25,753 (17,941), 32,301 (19,592), 37,300 (21,743), and 42,119 (25,692).

### Verification of the heart failure detection algorithm

Table 2 presents the performance characteristics derived from cross-validating the heart failure detection algorithms (defined in Table 1) against the reference standard set, i.e., the 1042 manually labeled inpatients, in whom 222 subjects (21%) were identified by the expert to suffer from heart failure.

**Table 2:** Performance of automated heart failure detection algorithms versus reference standard

Algorithm		*N*	Ref. HF+ *n* = 222	Ref. HF− *n* = 820	Precision (%)	Sensitivity (%)	F1 score (%)
*M*_ICD_	HF+	117	110 (TP)	7 (FP)	94	50	65
(for comparison)	HF−	925	112 (FN)	813 (TN)			
*M*_Expert_	HF+	253	193 (TP)	60 (FP)	76	87	81
(expert specified)	HF−	789	29 (FN)	760 (TN)			
*A*_Precision_	HF+	140	134 (TP)	6 (FP)	96	60	74
(precision optimized)	HF−	902	88 (FN)	814 (TN)			
*A*_Sensitivity_	HF+	286	204 (TP)	82 (FP)	71	92	80
(sensitivity optimized)	HF−	756	18 (FN)	738 (TN)			
*A*_F1_	HF+	209	186 (TP)	23 (FP)	89	84	86
(F1 score optimized)	HF+	833	36 (FN)	797 (TN)			

*Ref* reference standard defined by a heart failure specialist inspecting the documents, *HF+* heart failure present, *HF−* heart failure absent, *TP* true positive, *FP* false positive, *FN* false negative, *TN* true negative. Precision: TP/(TP + FP), sensitivity: TP/(TP + FN), F1: 2 × (precision × sensitivity)/(precision + sensitivity). For details refer to “Methods”

The algorithm that was solely based on ICD codes (*M*_ICD_) resulted in a good precision of 94%, but a low sensitivity of 50%, and a F1 score of 65%. The low sensitivity illustrates the low share of patients with heart failure detected by this algorithm. The missing 6% to a precision of 100% were caused by seven out of the 1042 patients who had a heart failure-related ICD diagnosis, but were not labeled to have heart failure. The expert-specified heart failure algorithm (*M*_Expert_) improved the detection rate and resulted in a precision of 76%, a sensitivity of 87%, and a F1 score of 81%. Divergent conclusions between the algorithm and the reference standard were found in 89 cases, with 60 patients mistakenly classified to have heart failure and 29 patients mistakenly classified to not have heart failure.

Since the manually defined algorithms resulted in low scores, the algorithm *M*_Expert_ was refined further. Three algorithms were developed and tested, each optimizing certain aspects of diagnostic accuracy: *A*_Precision_ aimed to increase the reliability of the classification as heart failure patient (reduced false positives), *A*_Sensitivity_ aimed to reduce the number of patients not classified as heart failure patient (reduced false negatives), and *A*_F1_ aimed for an overall improved accuracy of the classification as heart failure patient (balanced precision and sensitivity). The algorithm with the highest F1 score (i.e. *A*_F1_) resulted in a precision of 89%, a sensitivity of 84%, and an F1 score of 86%. The missing 14% to an F1 score of 100% was caused by 59 false matches that originated from “borderline cases” with limited or unclear textual information that opened more room for interpretation and misclassification for both computer and expert. Some errors were the result of missing data in the DWH, e.g., missing LVEF values or terms that indicate negations of heart failure in the discharge letter.

### Prevalence of heart failure

**Fig. 1:**
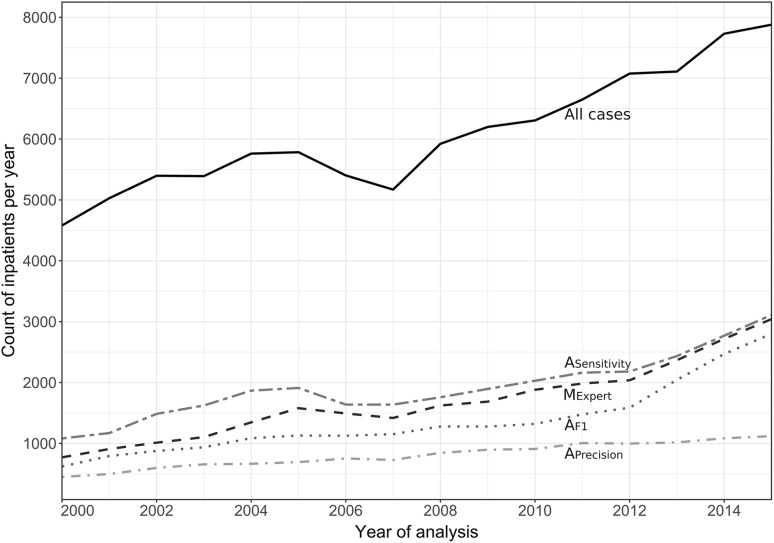
Count of inpatients within the Department of Medicine I in the years 2000–2015. The solid line indicates all patients; each patient is counted once per year. Intermittent lines represent patients with heart failure identified using different automated heart failure detection algorithms: *M*_Expert_ originates from the variable set pre-specified by the clinical expert; *A*_Precision_ optimizes count of false positives; *A*_Sensitivity_ optimizes count of false negatives; *A*_F1_ optimizes overall accuracy (for details refer to“Methods”)

Figure 1 illustrates the annual frequencies of all inpatients of the Department of Medicine I with a discharge letter and the subgroup of patients with heart failure identified by the automated algorithms described in Table 1. Across the entire period, *A*_F1_ identified 18,167 unique patients with heart failure. In the year 2000, the count of patients with heart failure started at *n* = 620 and showed an average annual gain of 9.3% over the entire period. After the year 2012, the annual gain appeared to accelerate from 7.4% before 2012 to 17.1% thereafter. By contrast, the average annual gain of all inpatients was 3.4%. The application of *A*_Precision_ and *A*_Sensitivity_ resulted in 10,786 and 25,084 patients identified with heart failure, respectively.

Several patients were treated multiple times over the years, which resulted in sums of unique patients per year reported in Fig. 1 that were higher than the above-reported sum of unique patients of all years. This included 3115 unique patients with 4583 heart failure-related re-hospitalizations after an initial heart failure-related hospitalization within the entire period. A characterization of inpatients with heart failure identified by the application of *A*_F1_ is presented in Table 3 for the four 4-year periods from 2000 to 2015, grouped by age and sex.

**Table 3:** Frequencies of all patients with heart failure identified by the *A*_F1_ algorithm by age group, gender and the 4-year periods (each with unique patients per time period)

	All patients with heart failure	Period			
		2000–2003	2004–2007	2008–2011	2012–2015
	*n* = 18,167	*n* = 3100	*n* = 4244	*n* = 4905	*n* = 8197
Sex, *n* (%)					
Male	10,636 (59)	1772 (57)	2466 (58)	2929 (60)	4945 (60)
Female	7531 (41)	1328 (43)	1778 (42)	1976 (40)	3252 (40)
Age category, *n* (%)					
≤ 45 years	702 (4) [67% male]	108 (3) [67% male]	173 (4) [70% male]	156 (3) [68% male]	301 (4) [64% male]
46–54 years	1294 (7) [74% male]	167 (5) [72% male]	287 (7) [76% male]	317 (6) [73% male]	589 (7) [75% male]
55–64 years	2766 (15) [73% male]	474 (15) [73% male]	571 (13) [77% male]	637 (13) [74% male]	1268 (15) [70% male]
65–74 years	5169 (28) [67% male]	956 (31) [65% male]	1262 (30) [67% male]	1311 (27) [68% male]	2062 (25) [68% male]
> 74 years	9163 (50) [51% male]	1431 (46) [46% male]	2004 (47) [47% male]	2540 (52) [52% male]	4052 (50) [54% male]

Data are count (percent). Inpatients with heart failure subdivided by sex and age categories. Some patients were admitted more than once; on average, one patient contributed 1.8 patient cases

**Fig. 2:**
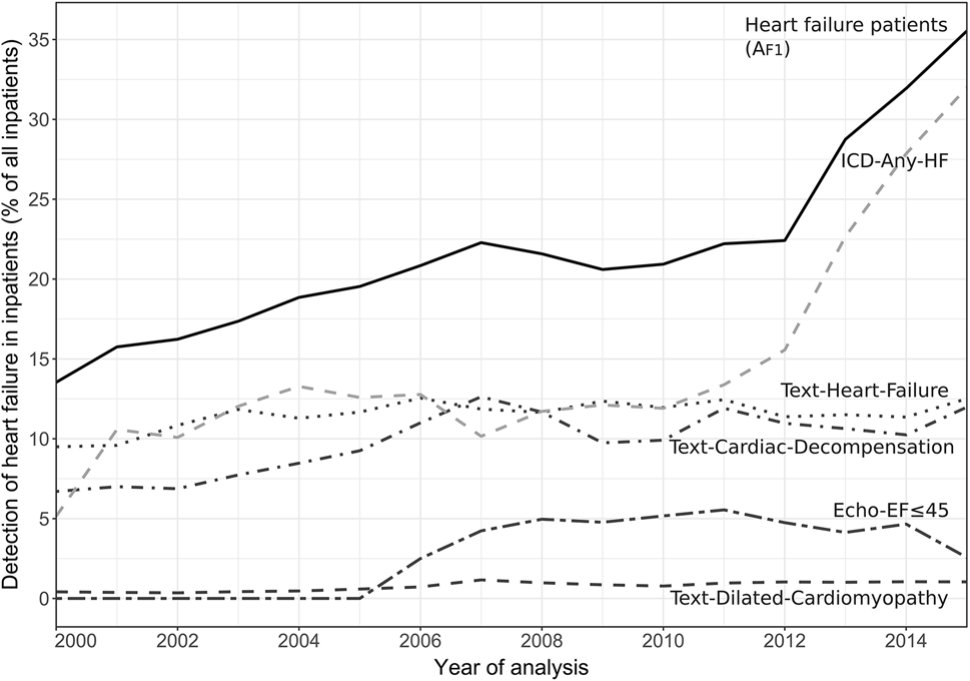
Detection of heart failure in inpatients using different approaches (percentage of all inpatients). The solid line indicates the prevalence detected when applying the automated algorithm *A*_F1_ (for details refer to “Methods”). Intermittent lines indicate detection using ICD codes or other information tags that dominantly contributed to the detection of heart failure. Each patient entered analysis only once per year; if patients attended the hospital multiple times, the first case of each patient per year was used

Each search term of the detection algorithm *A*_F1_ contributed with varying impact to the identification of heart failure. In the reference standard, the largest contributions emerged from “Text-Heart-Failure” (59% capture rate), “ICD-Any-HF” (56%), and “Text-Cardiac-Decompensation” (53%). Further important contributors were “Echo-EF ≤ 45” (24%) and “Text-Systolic-Failure” (4%). In the case of “ICD-Any-HF”, for example, this means that 44% of all patients with heart failure did not have an ICD code indicative of heart failure. The contribution of the individual search terms to the overall analysis varied substantially over the years, as presented in Fig. 2. This illustration presents the search terms of the first heart failure related hospitalization per patient and year. Noteworthy is the relatively small contribution of the term LVEF ≤ 45% from echocardiography, although echocardiography was frequently performed: in 58% of patients on cardiologic ward and 29% of patients on other wards of internal medicine.

**Fig. 3:**
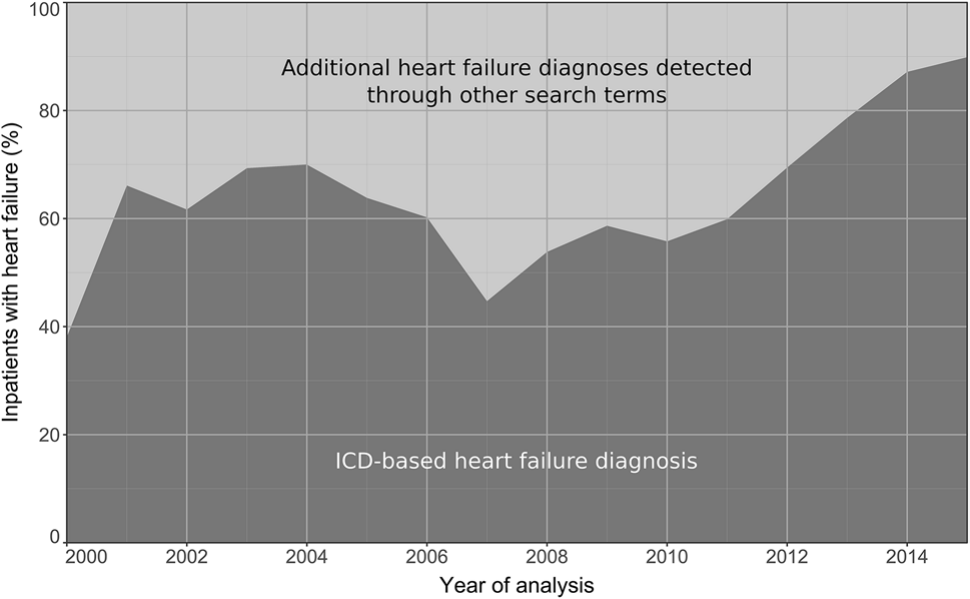
Detection of heart failure via related ICD codes (dark gray) and the additional detection through other search terms* (light gray), in inpatients with heart failure across the entire sampling period (years 2000–2015). The percentage of patients found via selective ICD code search increased in recent years, which might be explained by the foundation of the Comprehensive Heart Failure Center Würzburg in the year 2010, i.e., a facility devoted to the integration of research and care of patients with heart failure. *Executed via application of the automated algorithm A_F1_ (for details refer to “Methods”)

Figure 3 illustrates the contribution of ICD codes to the detection of heart failure in contrast to the additional contribution of other search terms (text/echo terms) over the entire period using the *A*_F1_ algorithm. Within the years 2000–2015, the overall share of patients with heart failure identified by ICD codes was 69% of the total sample of patients with heart failure, which means that 31% of patients with heart failure remained undetected throughout the entire period.

### Comorbidities and heart failure

We further analyzed, whether the comorbidity profile differed in subjects in whom presence of heart failure was identified via ICD codes versus subjects in whom heart failure was identified via additional sources of the DWH. Table 4 lists the most frequent comorbidities reported in the 18,167 patients with heart failure detected by the A_F1_ algorithm in the mutually exclusive subgroups “detected by ICD codes” or “detected by other search terms” (specific DWH interrogation other than ICD code). Reported comorbidities were identified by their respective ICD code. Patients with ICD-coded comorbidities more frequently also had an ICD code for heart failure. Of note, the subgroup identified without ICD codes appeared to have a slightly lower burden of comorbidity.

**Table 4:** Frequency of comorbidities in inpatients with heart failure, detected by ICD codes and additionally detected via data sources provided by the data warehouse

	All patients with heart failure^a^ *n* = 18,167	Heart failure detected by ICD codes *n* = 13,361	Heart failure detected on top via other sources *n* = 4806
Essential primary hypertension (I10)	11,335 (62.4)	8441 (63.2)	2894 (60.2)
Chronic ischemic heart disease (I20, I25)	9025 (49.7)	6801 (50.9)	2224 (46.3)
Heart valve disorders (I34-I39)	3495 (19.2)	3045 (22.8)	450 (9.4)
COPD (J44)	2874 (15.8)	2392 (17.9)	482 (10.0)
Acute myocardial infarction (I21)	2837 (15.6)	1990 (14.9)	847 (17.6)
Anemia (D60-D64)	1942 (10.7)	1529 (11.4)	413 (8.6)
Cardiomyopathy (I42)	1283 (7.1)	1041 (7.8)	242 (5.0)
Depression (F32, F33)	1031 (5.7)	848 (6.3)	183 (3.8)
Cerebral hemorrhage, infarction, stroke (I61, I63, I64)	387 (2.1)	313 (2.3)	74 (1.5)
Sleep apnea (G47.3)	287 (1.6)	240 (1.8)	47 (1.0)
Kidney failure (N19)	196 (1.1)	156 (1.2)	40 (0.8)

Comorbidities with ICD codes in descending order by prevalence in the total sample. Numbers are count (%)

*HF* heart failure, *COPD* chronic obstructive pulmonary disease

^a^Heart failure detection was done using the automated algorithm *A*_F1_ (for details refer to “Methods”)

## Discussion

The current analysis sheds new light on the magnitude of underestimation of heart failure prevalence in hospitalized patients. Identifying patients with heart failure from the hospital information system solely based on ICD-coded discharge diagnoses substantially underestimated the “true number” that could be gleaned after adding specific text searches and echocardiographic parameters to the search profile.

We observed a large degree of heart failure underestimation when using ICD codes only: within a single year it was up to 55% (average 31%) lower than the “true number” of heart failure. The last years of the analysis showed a trend towards better patient identification. The decreasing gap of underestimation became considerably smaller over time and indicates that coding strategies as diagnostic and therapeutic algorithms may indeed affect the “prevalence” of a disease. The detection gap came together with a marked increase in the absolute frequency of encoding ICD diagnoses for heart failure, starting with the year 2012 for inpatients. Furthermore, the percentage of patients with heart failure increased from about 15% in 2000 to about 35% in 2015. Reasons for such high proportions might be that we only included patients from the Medical Department I (hosting wards for intensive care, cardiology, pulmonology, endocrinology, nephrology), where heart failure is a frequent diagnosis, but also identified patients having heart failure as a secondary or tertiary diagnosis. Another explanation for these developments might be that the Comprehensive Heart Failure Center was founded at the Würzburg University Hospital in the year 2010, i.e., a facility devoted to the integration of research and care of patients with heart failure. This spurred numerous structural and research projects involving several hospital departments, led to a higher degree of awareness for the heart failure syndrome, and ultimately might not only have increased the count of patients with heart failure admitted to the hospital, but also improved the coding ratio.

Verifying the diagnosis of heart failure patients based on physician claims or hospital data has been attempted earlier [6, 14–21]. However, most of these studies focused on confirming or refuting the diagnosis of heart failure with the help of experts in subjects pre-identified via several variants of ICD codes, via study inclusion/exclusion criteria or manual screening. Subsequently, reported identification figures were fairly precise (i.e., yielded high precision). Frolova et al. for example aimed to verify ICD-based diagnosis of acute heart failure amongst patients admitted to the hospital with suspected acute heart failure [17] and found a precision of 93% (sensitivity of 76%) leading to an F1 score of 84%. In contrast, we aimed to identify “true heart failure” amongst all-comers, i.e., without an increased pre-test likelihood for the presence of such diagnosis. As expected, performance of the algorithm relying solely on ICD-based identification (= *M*_ICD_) was worse in our data set (F1 score 65%). There are few studies focusing on all-comers for detecting heart failure [15, 22, 23], all reporting lower F1 scores (82, 53–67, 80%, respectively) compared to our analysis (86%).

Applying text extraction methods to detect heart failure has rarely been attempted. Meystre et al. [24], for example focused on the information extraction of a few highly selected parameters (e.g., LVEF value and medication) from texts in contrast to an overall detection of heart failure. They utilized a pre-defined data set of heart failure patients in contrast to all-comers and, subsequently, received high F1 scores of up to 99% for single parameters (e.g., LVEF value). While interesting to demonstrate feasibility, such concepts do not mirror clinical reality. No related work was found utilizing information extraction to detect heart failure in all-comers.

Another major finding of the current study is that readily available information from the hospital information system considerably improves the identification of heart failure patients beyond the traditional identification via ICD codes. The option to enrich the search strategy by clinical variables supporting or denying the presence of heart failure is not new, but a variety of problems may impede its implementation: (1) the information is only selectively documented in clinical routine; (2) the desired information is stored in a non-structured format and appropriate data extraction tools are unavailable or unreliable; (3) the information is stored in a structured format but cannot be accessed for analysis (e.g., because it is stored in dedicated research data bases); (4) the quality of the stored data (structured or non-structured) is unreliable; (5) the information behind variables (meta data) is highly flexible but cannot be connected to the source data; (6) the individual patient and the corresponding cases of a patient (repeat hospitalizations) cannot reliably be discerned. Our approach utilized the hospital’s clinical DWH as described earlier [8–10] and integrated the full spectrum of digital information collected per patient in the hospital information system.

The most elaborated part in providing a DWH is the implementation of the data extract–transform–load (ETL) process to transfer data from the information systems to a unified database, which often—but not always—requires to consider local peculiarities depending on the available information systems. We implemented this process for most of the information stored within our systems, be it structured or unstructured. Our DWH query system utilizes a locally developed add-on [10] to provide text search functionality to DWH systems. This add-on could be instantaneously added as an extension to the often utilized i2b2 DWH system [25] or, with little extra work, to other similar DWH systems. Importantly, these tools were tested and optimized across their repeat utilization for various studies, including data validation against the primary systems after DWH extraction [9].

The combined use of these interfaces and generation of automated detection algorithms markedly improved the identification of patients with heart failure. We found better albeit still unsatisfactory accuracy when employing the algorithm based on “clinical information” alone (i.e., the algorithm *M*_Expert_). We therefore tested other, data-optimized algorithms, and observed another major improvement of heart failure detection: the algorithm *A*_F1_ optimized precision and sensitivity and yielded the overall best results. Importantly, our approach allowed to adjust and optimize the detection algorithm for different scenarios or use cases, e.g., to identify potential study participants via the algorithm (and thus enabling a study nurse to fine-tune the results) *A*_Sensitivity_ might yield best results. For the scenario of a post hoc analysis, *A*_F1_ or *A*_Precision_ might be the preferred solutions. Interestingly, the NT-proBNP queries “Lab-NT-proBNP ≥ 1000” and “Lab-NT-proBNP ≥ 3000” (see Table 1) were not selected by the permutation analysis for any algorithm. This may be explained by the collinearity contained in other terms indicative for heart failure; e.g., for the *A*_F1_-algorithm: “Echo-EF ≤ 45”, “ICD-Any-HF”, “Text-Heart-Failure”, “Text-Cardiac-Decompensation”, and “Text-Systolic-Failure”. We also considered using Framingham heart failure signs and symptoms [26] for detection of heart failure (see [27, 28]) either alone or in combination with borderline echocardiographic data, but were unsuccessful in demonstrating superior precision and sensitivity.

Our analyses support the notion that comorbidities of heart failure may also affect coding practices for heart failure. When comparing the presence of common comorbidities with the detection of heart failure via ICD-based versus alternative approaches, the differences where highly significant for almost all conditions. Interestingly, a sizeable proportion of patients with heart failure received an ICD code for the respective comorbidity, but not the ICD code for heart failure itself. This might indicate that heart failure was not at the focus of their hospitalization visit and not a dominant contributor from the reimbursement perspective. From a health policy perspective this means that many patients with heart failure as a concomitant condition leave the hospital without being reported to statutory data banks as heart failure patients. This not only adds to the detection gap, but also constitutes a major information gap for care providers after hospital discharge who play a key role in the treatment of heart failure in Germany [29].

### Limitations

A limitation of this study is that the reference standard was only defined by a single cardiologist with long-standing experience in heart failure instead of multiple experts. The count of true heart failure patients may vary considerably depending on the care setting, the type of catchment area, and numerous other influencing factors. Hence, absolute counts are likely not directly comparable between hospitals. Similarly, the successful implementation of adapted detection algorithms needs to be confirmed before our results may become generalizable to other hospitals, both in Germany as internationally.

## Conclusions

Coded discharge diagnoses substantially underestimate the number of heart failure patients compared to the added information available within discharge letters and echocardiographic reports. Therefore, statistics about heart failure solely based on ICD codes might be misleading. The degree of underestimation might vary substantially across case types (inpatients versus outpatients) and the course of subsequent years. The latter might be influenced by internal factors, e.g., improved coding practices, and/or external factors, e.g., the set up of specialized centers as the Comprehensive Heart Failure Center Würzburg.

## References

[1] Federal Statistical Office. http://www.gbe.de. Accessed Mar 2017

[2] Asakura K, Ordal E (2012). Is your clinical documentation improvement program compliant?. Health Financ Manag.

[3] Pourasghar F, Malekafzali H, Koch S, Fors U (2008). Factors influencing the quality of medical documentation when a paper-based medical records system is replaced with an electronic medical records system: an Iranian case study. Int J Technol Assess Health Care.

[4] Hall MJ, Levant S, DeFrances CJ (2012) Hospitalization for congestive heart failure: United States, 2000–2010, NCHS Data Brief (108), pp 1–8 23102190

[5] Schellenbaum GD, Heckbert SR, Smith NL, Rea TD, Lumley T, Kitzman DW, Roger VL, Taylor HA, Psaty BM (2006). Congestive heart failure incidence and prognosis: case identification using central adjudication versus hospital discharge diagnoses. Ann Epidemiol.

[6] Saczynski JS, Andrade SE, Harrold LR, Tjia J, Cutrona SL, Dodd KS, Goldberg RJ, Gurwitz JH (2012). A systematic review of validated methods for identifying heart failure using administrative data. Pharmacoepidemiol Drug Saf.

[7] Störk S, Handrock R, Jacob J, Walker J, Calado F, Lahoz R, Hupfer S, Klebs S (2017). Epidemiology of heart failure in Germany: a retrospective database study. Clin Res Cardiol.

[8] Fette G, Ertl M, Wörner A, Kluegl P, Störk S, Puppe F (2012) Information extraction from unstructured electronic health records and integration into a Data Warehouse, Lecture notes in informatics (LNI) vol 208, pp 1238–1252

[9] Kaspar M, Ertl M, Fette G, Dietrich G, Toepfer M, Angermann C, Störk S, Puppe F (2016). Data linkage from clinical to study databases via an R data warehouse user interface. Experiences from a large clinical follow-up study. Methods Inf Med.

[10] Dietrich G, Ertl M, Fette G, Kaspar M, Krebs J, Mackenrodt D, Störk S, Puppe F (2017). Extending the query language of a data warehouse for patient recruitment. Stud Health Technol Inform.

[11] Toepfer M, Fette G, Beck PD, Klügl P, Puppe F (2014) Integrated tools for query-driven development of light-weight ontologies and information extraction components. In: Ide N, Grivolla J, (eds) Proceedings of the workshop on open infrastructures and analysis frameworks for HLT; Association for Computational Linguistics and Dublin City University, pp 83–92

[12] Aho AV (1990). Algorithms for finding patterns in strings; handbook of theoretical computer science, volume A: algorithms and complexity.

[13] R Development Core Team (2008) R: a language and environment for statistical computing. R Foundation for Statistical Computing, Vienna. ISBN 3-900051-07-0. http://www.R-project.org. Accessed 13 Apr 2018

[14] Quach S, Blais C, Quan H (2010). Administrative data have high variation in validity for recording heart failure. Can J Cardiol.

[15] Schultz SE, Rothwell DM, Chen Z, Tu K (2013). Identifying cases of congestive heart failure from administrative data: a validation study using primary care patient records. Chronic Dis Inj Can.

[16] Agarwal SK, Wruck L, Quibrera M, Matsushita K, Loehr LR, Chang PP, Rosamond WD, Wright J, Heiss G, Coresh J (2016). Temporal trends in hospitalization for acute decompensated heart failure in the United States, 1998–2011. Am J Epidemiol.

[17] Frolova N, Bakal JA, McAlister FA, Rowe BH, Quan H, Kaul P, Ezekowitz JA (2015). Assessing the use of international classification of diseases-10th revision codes from the emergency department for the identification of acute heart failure. JACC Heart Fail.

[18] Rosenman M, He J, Martin J, Nutakki K, Eckert G, Lane K, Gradus-Pizlo I, Hui SL (2014). Database queries for hospitalizations for acute congestive heart failure: flexible methods and validation based on set theory. J Am Med Inform Assoc.

[19] Alqaisi F, Williams LK, Peterson EL, Lanfear DE (2009). Comparing methods for identifying patients with heart failure using electronic data sources. BMC Health Serv Res.

[20] Loehr LR, Agarwal SK, Baggett C, Wruck LM, Chang PP, Solomon SD, Shahar E, Ni H, Rosamond WD, Heiss G (2013). Classification of acute decompensated heart failure: an automated algorithm compared with a physician reviewer panel: the Atherosclerosis Risk in Communities study. Circ Heart Fail.

[21] Khand AU, Shaw M, Gemmel I, Cleland JG (2005). Do discharge codes underestimate hospitalisation due to heart failure? Validation study of hospital discharge coding for heart failure. Eur J Heart Fail.

[22] Quan H, Parsons GA, Ghali WA (2002). Validity of information on comorbidity derived from ICD-9-CCM administrative data. Med Care.

[23] Quan H, Li B, Saunders LD, Parsons GA, Nilsson CI, Alibhai A, Ghali WA (2007). Assessing validity of ICD-9-CM and ICD-10 administrative data in recording clinical conditions in a unique dually coded database. Health Serv Res.

[24] Meystre SM, Kim Y, Gobbel GT, Matheny ME, Redd A, Bray BE, Garvin JH (2017). Congestive heart failure information extraction framework for automated treatment performance measures assessment. J Am Med Inform Assoc.

[25] Murphy SN, Weber G, Mendis M, Chueh HC, Churchill S, Glaser JP, Kohane IS (2010). Serving the enterprise and beyond with informatics for integrating biology and the bedside (i2b2). J Am Med Inform Assoc.

[26] McKee PA, Castelli WP, McNamara PM, Kannel WB (1971). The natural history of congestive heart failure: the Framingham study. N Engl J Med.

[27] Byrd R, Steinhubl S, Sun J, Ebadollahi S, Stewart W (2014). Automatic identification of heart failure diagnostic criteria, using text analysis of clinical notes from electronic health records. Int J Med Inform.

[28] Vijayakrishnan R, Steinhubl S, Ng K, Sun J, Byrd R, Daar Z, Williams B, Defilippi C, Ebadollahi S, Stewart W (2014). Prevalence of heart failure signs and symptoms in a large primary care population identified through the use of text and data mining of the electronic health record. J Cardiac Fail.

[29] Störk S, Handrock R, Jacob J, Walker J, Calado F, Lahoz R, Hupfer S, Klebs S (2017). Treatment of chronic heart failure in Germany: a retrospective database study. Clin Res Cardiol.

